# Characteristics of Tungsten Prepared by Hot Pressing at High Pressure

**DOI:** 10.3390/ma18235265

**Published:** 2025-11-21

**Authors:** Jiří Matějíček, Monika Vilémová, Andrii Rednyk, Hynek Hadraba, Zdeněk Chlup, František Lukáč, Romain Génois, Jakub Klečka

**Affiliations:** 1 Institute of Plasma Physics of the Czech Academy of Sciences, U Slovanky 1a, 18200 Praha, Czech Republic; vilemova@ipp.cas.cz (M.V.); lukac@ipp.cas.cz (F.L.); genois@ipp.cas.cz (R.G.); klecka@ipp.cas.cz (J.K.); 2 Institute of Physics of Materials of the Czech Academy of Sciences, Žižkova 22, 61600 Brno, Czech Republic; hadraba@ipm.cz (H.H.); chlup@ipm.cz (Z.C.)

**Keywords:** tungsten, hot pressing, plasma facing materials, powder metallurgy

## Abstract

Tungsten is a prime candidate material for the plasma-facing components of fusion reactors, thanks to its high melting point, high temperature strength, good thermal conductivity, high erosion resistance, etc. Yet, it has some limitations, mainly its brittle nature, difficulty of machining, and propensity to recrystallize at elevated temperatures. Among the approaches to the improvement of particular properties are alloying, dispersion strengthening, thermomechanical processing, and modifications to the sintering process. This study explores the possibility of combining fine powder size with ultra-high pressure to achieve significant densification at moderate temperatures during hot pressing. Two powder sizes and a range of temperatures from 1000 to 2000 °C were used, and their effects were observed. The resulting tungsten compacts were characterized for their microstructure, density, and mechanical and thermal properties. The high pressure enabled substantial densification already at relatively low temperatures, thanks to the plastic deformation of the powder particles. A significant degree of sintering, as manifested by the microstructural and property evolution, occurred however only at higher temperatures. The compacts exhibited brittleness, calling for further optimization of the method.

## 1. Introduction

Tungsten (W) is a refractory metal with exceptional properties, including the highest melting point of all metals (3410 °C), a high density (19.3 g/cm^3^), excellent thermal conductivity, and superior mechanical strength at elevated temperatures. These characteristics make tungsten an ideal candidate for extreme environment applications, such as in fusion reactor components [1,2,3] and aerospace technologies [4]. However, its sintering behavior presents significant challenges due to its high melting point and poor sinterability. Impurities such as Ni, Fe, or additions that form oxide or carbide dispersions (Ti, Y, Zr) may enhance sintering, and such an alloyed tungsten may require a lower sintering temperature to achieve a theoretical density above 95%. However, for pure tungsten, conventional sintering methods require extreme temperatures (>2000 °C) and long dwell times [5], which promote excessive grain growth and degrade the mechanical properties. To overcome these limitations, high-pressure sintering (HPS) techniques have emerged as a promising solution. The key mechanism that enhances densification during high-pressure sintering is the activation of pressure-induced plastic deformation and enhanced diffusional creep, which accelerate particle rearrangement and pore closure. As a result, lower sintering temperatures may be needed, and thus, grain growth can be significantly suppressed, enabling the preservation of fine-grained microstructures and superior mechanical properties.

Bulk tungsten is generally considered a brittle material, mainly because the ductile-to-brittle transition temperature for 99.95% pure, micron-sized tungsten typically ranges from 500 to 1000 °C [6]. This brittleness is largely attributed to the presence of grain boundaries, which represent the weakest regions in the microstructure. However, the plasticity of tungsten powder can differ significantly, particularly in the case of submicron- and micron-sized particles, which may be monocrystalline and therefore lack grain boundaries. In such cases, individual powder particles can exhibit plastic deformation at temperatures as low as 200 °C, leading to improved powder packing and particle rearrangement even at temperatures below the onset of sintering densification.

Although the number of studies conducted on pure tungsten using high-pressure sintering (HPS) remains limited, they already demonstrate clear advantages over conventional sintering, particularly in promoting rapid densification, minimizing grain growth, and enhancing mechanical properties. Across various HPS approaches (Table 1), relative densities in the range of 94–98.5% of the theoretical value are typically achieved, often within seconds to a few minutes of sintering time. These high densities are obtained at significantly lower temperatures, generally between 1200 °C and 1600 °C. However, HPS also presents several challenges, including the risk of crack formation due to internal stresses generated by rapid densification under high pressure.

In the present study, we investigate the combined effect of micron and submicron tungsten powder and high pressure on the sintering behavior of tungsten. While previous studies have primarily focused on the achieved densities and hardness, our aim is to contribute to the understanding of high-pressure sintering effects by placing particular emphasis on the microstructural evolution induced by high-pressure sintering. Additionally, we examine the thermal and tensile properties, which can offer further insight into the quality of interparticle bonding achieved at reduced sintering temperatures.

## 2. Materials and Methods

### 2.1. Materials and Sample Preparation

For the preparation of the samples, pure W powders of two average particle sizes were used—around 0.4 µm (designated MP02S) and around 2 µm (MP20S), both produced by Global Tungsten and Powders (Bruntál, Czech Republic). According to the manufacturer’s specification, the 0.4 µm powder contained 0.28 wt.% oxygen and the 2 µm powder 0.034 wt.% oxygen. The powders were consolidated by hot pressing at Bonar (Šumperk, Czech Republic), at the following conditions: pressure of 6 GPa, sintering time of 28 min, sintering temperatures of 1000, 1400, 1800, and 2000 °C, resulting in eight sample variants (two powders times four temperatures). A proprietary die configuration was used to allow the achievement of such high pressure. Disks of 19 mm diameter and 8 mm thickness were produced. 

### 2.2. Characterization

The as-sintered disks were cut up into several smaller samples for specific characterizations by electric discharge machining. Microstructural observations were carried out on metallographically polished cross-sections, as well as on fracture surfaces, using a scanning electron microscope (SEM) EVO MA15 (Carl Zeiss SMT, Oberkochen, Germany) and Apreo 2 S LoVac (Thermo Fisher Scientific, Brno, Czech Republic) with 100 mm^2^ Ultim Max EDS detector (Oxford Instruments, High Wycombe, UK) (for high-resolution microstructural analysis). For better visualization of the microstructure, the polished samples were also etched using a solution prepared by mixing 9 mL of hydrogen peroxide (90%) with 1 mL of potassium hydroxide solution (1 mol L^−1^). Etching was performed by wiping the cross-sections five times with solution-soaked tissues. Subsequently, the samples were rinsed with distilled water followed by ethanol to remove residual etchant and were then dried in air. Grain sizes were evaluated from these images using the line intercept method. X-ray diffraction (XRD) was carried out on the fracture surfaces using a D8 Discover diffractometer (Bruker, Karlsruhe, Germany) to quantify the crystalline sizes and microstrains; these were determined by a full-profile Rietveld refinement using the TOPAS 5 software. For tensile testing, flat dog-bone-shaped tensile specimens with a nominal cross-section of 1.5 mm × 1.5 mm and a gauge length of 5.2 mm were machined from the sintered compacts by electro-discharge machining. Their surfaces were subsequently polished to a mirror finish using 1 μm diamond paste. Tensile tests were then performed at 500 and 800 °C with a crosshead speed of 0.1 mm/min, in accordance with the ISO 6892-1:2018 standard [11] (Kappa, Zwick/Roell, Ulm, Germany). Thermal conductivity was determined at room temperature, 200, 400, and 600 °C by the flash method, using an LFA 1000 apparatus (Linseis, Selb, Germany). Microhardness measurements were performed on the polished cross-sections using a Q10A+ universal hardness tester (Qness, Golling an der Salzach, Austria) at 500 gf (HV 0.5) load and with 10 indentations on each sample. Density was determined by the Archimedean method (water immersion) on the thermal conductivity samples.

## 3. Results

**Figure 1:**
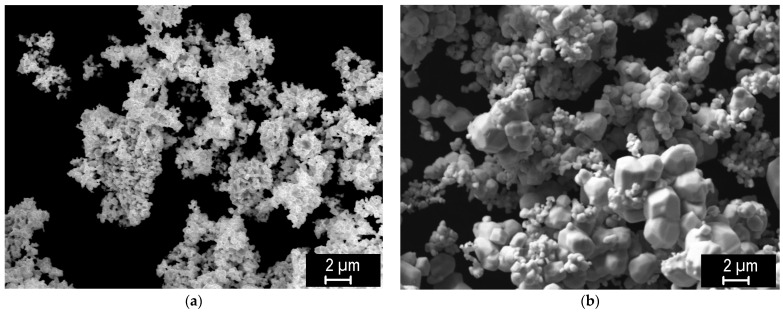
Overview of the powders: 0.4 µm (**a**) and 2 µm (**b**).

The two starting powders are shown in Figure 1. While the finer powder has submicron grains corresponding to the nominal size (0.4 µm), the coarser powder (average size ~2 µm) exhibited a range of sizes from above 2 µm down to submicron particles comparable to the finer powder. Due to the small size, both powders showed a tendency to agglomerate.

**Figure 2:**
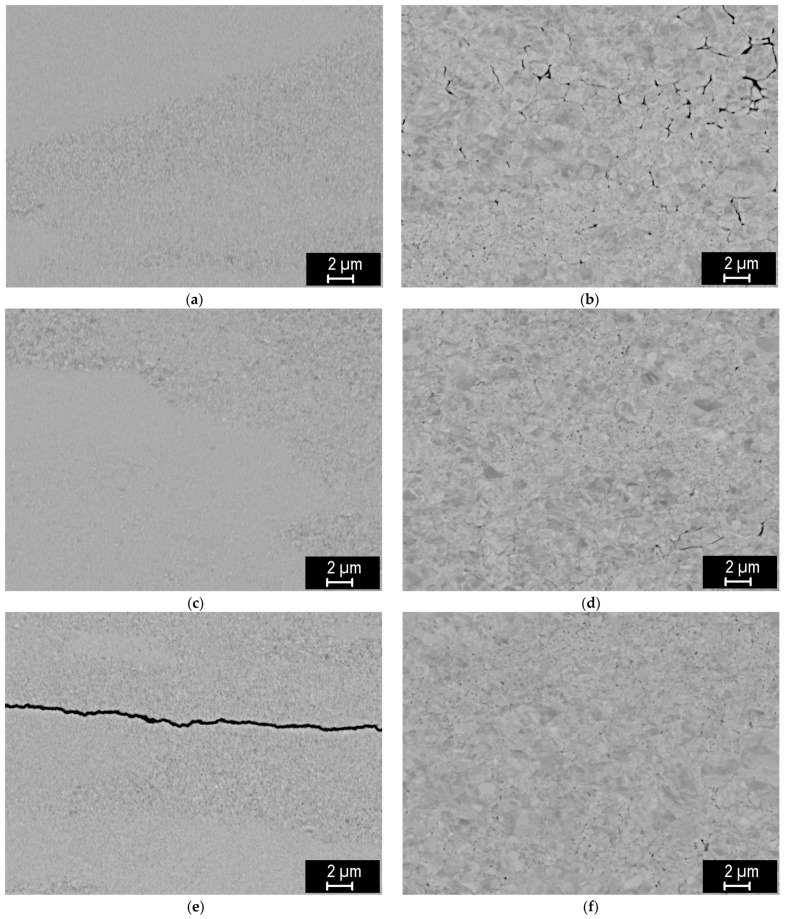

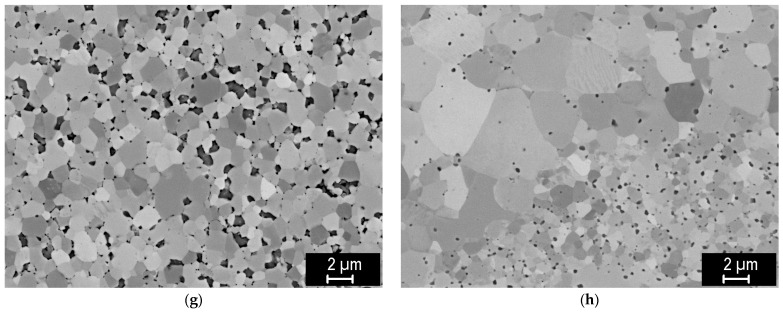
Polished cross-sections of hot-pressed W compacts from the 0.4 µm powder (left) and 2 µm powder (right), (**a**,**b**) sintered at 1000 °C; (**c**,**d**) 1400 °C; (**e**,**f**) 1800 °C; (**g**,**h**) 2000 °C.

SEM images of the polished cross-sections of the sintered compacts are shown in Figure 2. At lower temperatures, there is very low contrast between individual grains, possibly due to plastic deformation at the extremely high pressure. It is only at the highest sintering temperature that the typical microstructure of sintered tungsten is observed, with grain sizes on the order of microns. For the 0.4 µm powder, grain sizes are typically 2 µm or less, while for the 2 µm powder, they have a wider distribution, ranging from ~1 µm to several µm. For the 0.4 µm powder, pores are typically found at grain boundaries, while for the 2 µm powder, both inter- and intragranular pores can be seen, indicating a higher degree of sintering in this case (see below) [12]. For the lower sintering temperatures, individual pores could not be discerned, apart from the signs of incomplete sintering at the lowest temperature and the 2 µm powder. In some cases, cracking of the compacts was observed, either in the form of one major crack (cases e and g) or several finer cracks (case d). This was likely caused by the extremely high pressure, possibly combined with non-uniform sintering and the brittle nature of the material. The images also indicate a spatially inhomogeneous degree of sintering.

**Figure 3:**
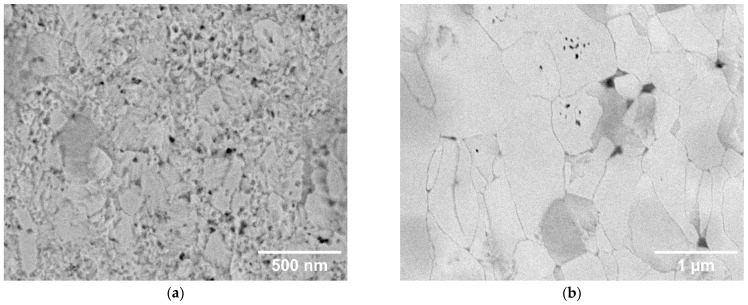

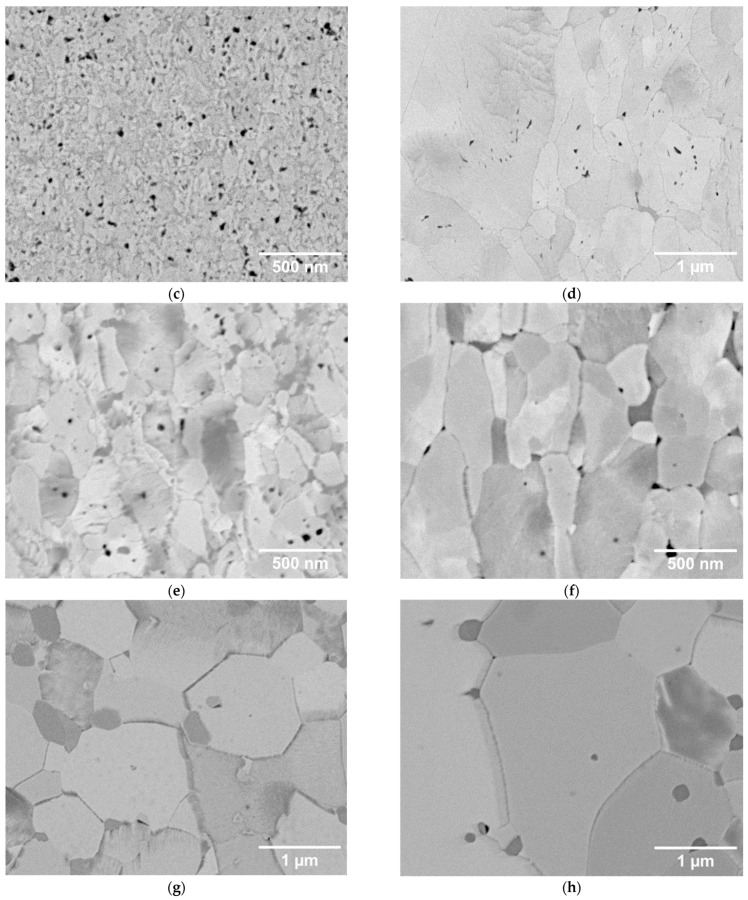
Polished and etched cross-sections of hot-pressed W compacts from the 0.4 µm powder (left) and 2 µm powder (right), (**a**,**b**) sintered at 1000 °C; (**c**,**d**) 1400 °C; (**e**,**f**) 1800 °C; (**g**,**h**) 2000 °C.

For a better visualization of the grain structure, the etched samples are shown in Figure 3. For the compacts made from the 0.4 µm powder, ultrafine grains are barely discernible for the 1000 and 1400 °C sintering temperatures. It is only at 1800 °C that submicron grains comparable to the starting powder size are revealed, and notably larger grains (above 0.4 µm) can be seen at 2000 °C. For the compacts made from the 2 µm powder, grain boundaries are well visible for all sintering temperatures. For the 1000–1800 °C temperatures, the grains are in the µm range or slightly below. They clearly show a deformed shape, demonstrating plastic deformation due to the high pressure already from the lowest temperatures. At a 2000 °C sintering temperature, significantly larger grains are observed (several µm). They also exhibit an equiaxial shape, indicating that the sintering-induced grain growth has superseded the initially deformed boundaries from the high pressure. A similar equiaxial morphology is seen for the 0.4 µm powder sintered at the highest temperature. Elemental analysis by energy-dispersive spectroscopy (EDS) has shown higher oxygen concentration at the grain boundaries; at the highest temperatures, it apparently precipitated in the form of nanometric oxide particles (small dark particles at the grain boundary junctions visible in Figure 3g,h).

**Figure 4:**
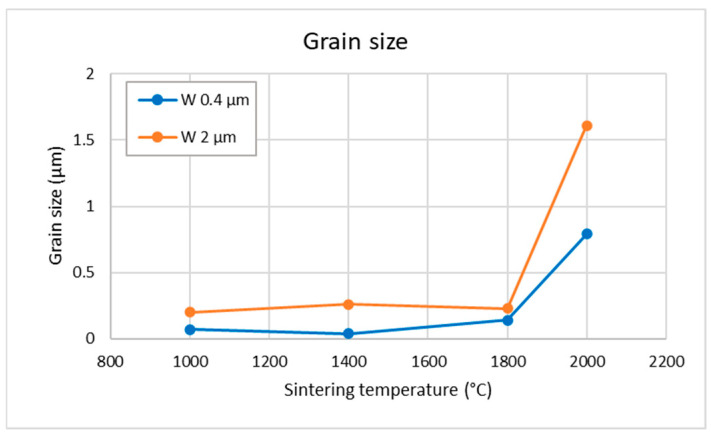
Grain size evaluated from the microscopy images. The typical coefficient of variation was about 10% for the 0.4 µm samples and 19% for the 2 µm samples.

Figure 4 shows the grain sizes evaluated from the microscopy images. The quantitative trends confirm the observations described above. It should be noted that the grain boundaries were difficult to discern for the lower temperatures in the 0.4 µm samples and that there was a noticeable spatial variation in grain size in the samples. 

**Figure 5:**
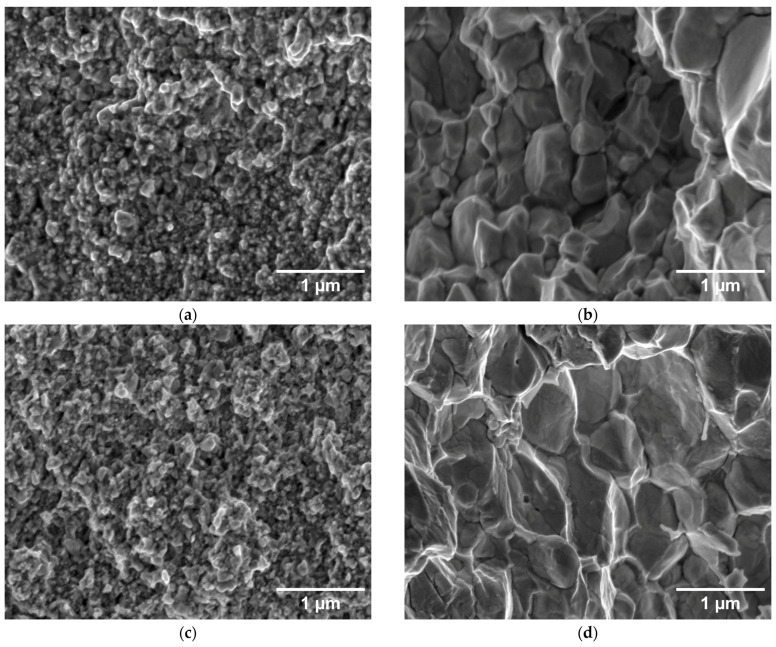

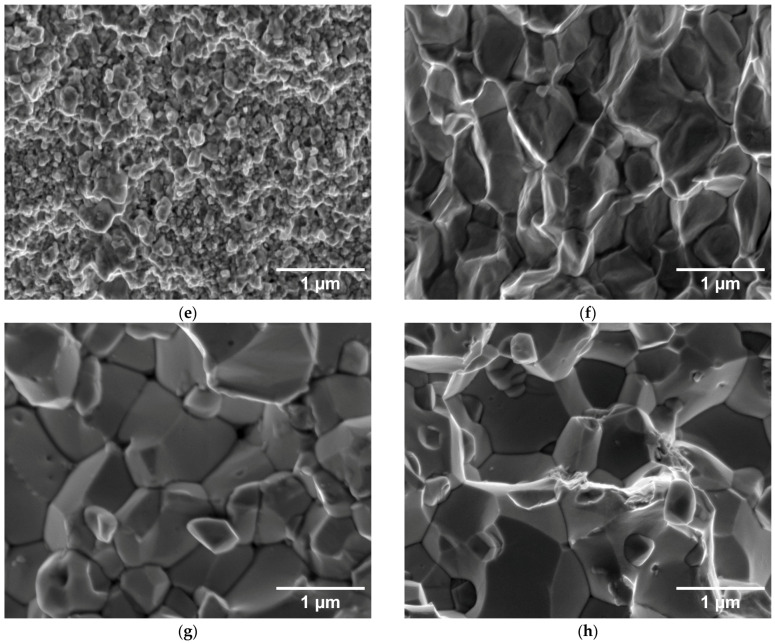
Fracture surfaces of hot-pressed W compacts from the 0.4 µm powder (left) and 2 µm powder (right), (**a**,**b**) sintered at 1000 °C; (**c**,**d**) 1400 °C; (**e**,**f**) 1800 °C; (**g**,**h**) 2000 °C.

The fracture surfaces of the corresponding samples are shown in Figure 5. For the 1000, 1400, and 1800 °C sintering temperatures, mostly ultrafine grains are observed, roughly comparable to the initial powders, which indicates a limited degree of sintering. It is only at the highest sintering temperature of 2000 °C that a noticeable grain growth can be seen, in agreement with the observations on the polished sections. On the compacts sintered from the larger powder, deformed grains are again observed at 1000–1800 °C, while the shape changes to equiaxial at 2000 °C, in agreement with the cross-sectional observations.

**Figure 6:**
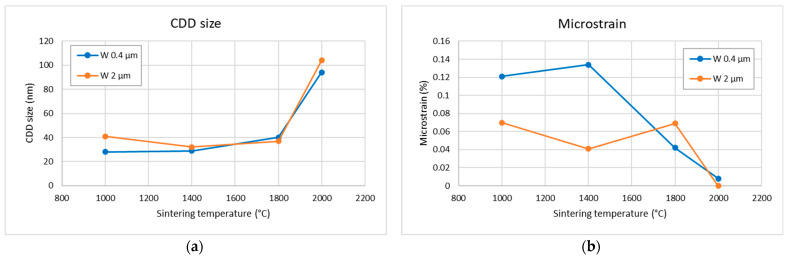
X-ray diffraction results, (**a**) size of coherently diffracting domains (CDDs); (**b**) microstrain. Typical coefficients of variation were about 3% for CDD size and 8% for microstrain.

Alternative information about the evolution of the crystallite size can be obtained from the XRD results. Figure 6 shows the size of the coherently diffracting domains as a function of the sintering temperature. For the lower temperatures, it remains roughly constant but increases significantly only at the 2000 °C sintering temperature. Similarly, the microstrain also decreases significantly at the highest sintering temperature. XRD did not detect any carbides or oxides. The XRD patterns are provided in the supplemental file.

**Figure 7:**
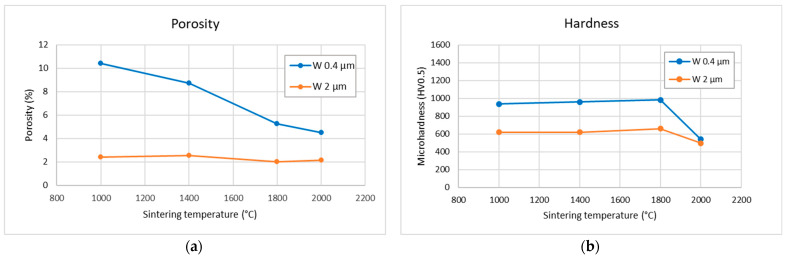
Porosity (**a**) and microhardness (**b**) as functions of sintering temperature. Typical coefficients of variation were about 6% for porosity and 4% for hardness.

Figure 7a shows the evolution of porosity (determined from density measured by the Archimedean method, assuming pure W skeleton) as a function of sintering temperature for the two powders. Expectedly, the porosity of compacts sintered from the 0.4 µm powder decreases with sintering temperature. Somewhat surprisingly, the porosity of compacts made from the 2 µm powder is lower and does not show a strong variation with temperature. The higher porosity of the 0.4 µm compacts may be caused by the finer powder’s higher tendency to agglomeration, and thus less homogeneous settling of the powder in the die, which may lead to regions of higher porosity. The negligible changes in porosity in the 2 µm powder compacts agree well with the SEM observations in Figure 3. Microhardness measurements were also performed to obtain an additional qualitative measure of the degree of sintering. The results are shown in Figure 7b. The plots show nearly no trend with respect to the sintering temperature, except for a change at 2000 °C. The trends suggest a correlation with grain size, as predicted by the Hall-Petch relationship [13,14]. For one, the hardness is higher overall for compacts made from the finer powder, despite their generally lower degree of sintering. Furthermore, the hardness is slightly reduced for both powders at the highest sintering temperature, where the grains have grown to a discernible size.

**Figure 8:**
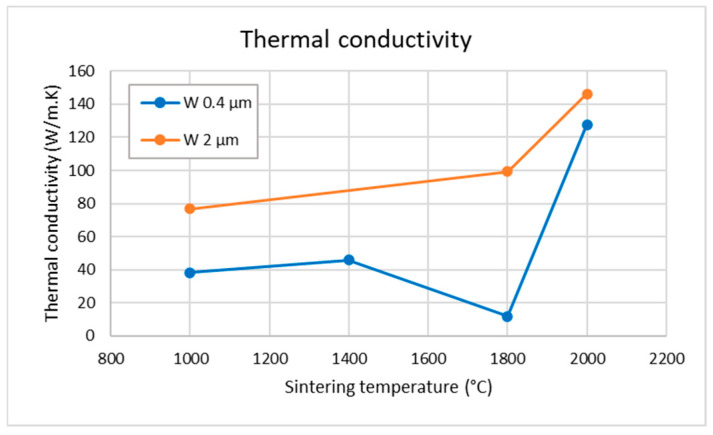
Thermal conductivity (measured at room temperature) as a function of sintering temperature. The value for the 2 µm sample at 1400 °C is missing, as the sample was damaged during preparation. The typical coefficient of variation was about 4%.

Figure 8 shows the thermal conductivity as a function of sintering temperature. The values are generally lower than those of fully dense W, and only increase significantly at the highest sintering temperature of 2000 °C, similarly to the other characteristics mentioned above. In agreement with the lower porosity, the 2 µm compacts show higher values than those from the 0.4 µm powder and approach those reported for fully dense W (160–170 W/m·K) [15,16] at the maximum sintering temperature. The reduced value for 0.4 µm and 1800 °C is likely caused by the cracks (cf. Figure 2e). The measurements at higher temperatures showed roughly similar values as at room temperature and principally the same trends with respect to individual samples.

**Figure 9:**
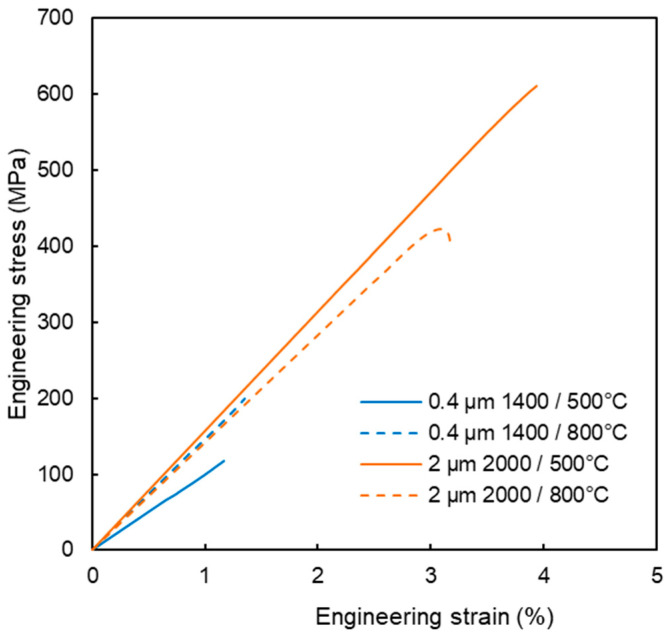
Stress–strain behavior of the hot-pressed compacts. The values 1400 and 2000 refer to the sintering temperatures, and 500 and 800 °C are the test temperatures. Samples from the 0.4 µm powder sintered at 1800 and 2000 °C were not tested because of the presence of macrocracks.

Tensile properties are illustrated in Figure 9. The tests were started at 800 and 500 °C, where ductile behavior could be most likely expected. For all samples, the stress increased linearly up to the point of fracture, reaching strains in units of %. Fracture surfaces (Figure 4) show dominantly intergranular fracture, i.e., along grain boundaries, without any marked signs of ductile behavior. This is clearly visible on the 2 µm powder compacts, but less discernible on the 0.4 µm compacts. Minor oxygen impurities, as detected by EDS, may have contributed to the weakening of the grain boundaries [17,18]. It might be noted that carbon diffusing into the samples from the graphite die presents a reducing environment [19,20]. Since all four tested samples exhibited brittleness, even at these elevated temperatures, it was decided not to test all variants at all temperatures.

## 4. Discussion of the Densification/Sintering Mechanisms

The densification behavior observed in this study suggests that the consolidation of tungsten under 6 GPa proceeds in two distinct stages: (i) particle plastic deformation, dominant up to 1800 °C, followed by (ii) thermally activated diffusional processes, which become active above 1400 °C.

At the lower sintering temperatures (1000–1400 °C), the plastically elongated grains observed in Figure 3 indicate that densification is driven predominantly by plastic deformation. This interpretation is consistent with the high microstrain values (Figure 6b), which start to relax at higher temperatures. Experimental observations confirm that tungsten of conventional purity can undergo plastic deformation already at several hundred °C [21]. In this early compaction stage, pore closure is therefore primarily mechanical rather than diffusional, which explains why relative density increases even when grain growth or boundary migration remains limited (see Figure 7a for 0.4 µm powder at 1000 and 1400 °C). For the 2 µm powder, the applied pressure results in near-maximum achievable density already at 1000 °C. However, the formation of interparticle bonding is required to achieve high mechanical strength (compare 0.4 µm vs. 2 µm in Figure 9).

A transition to diffusion-dominated sintering becomes clearly apparent above 1400 °C for the 0.4 µm powder and as an onset in the 2 µm powder. This agrees well with the known sintering behavior of tungsten, where this temperature range marks the beginning of the densification stage [12]. The activation of diffusional processes is evident from pronounced particle coarsening in the 0.4 µm samples (Figure 3e) and from the more subtle smoothing/rounding of fracture surface features in the 2 µm samples (Figure 5f). Above 1800 °C, equiaxed grains appear, the size of coherently diffracting domains increases, and microstrain further decreases (Figure 6), indicating the recovery of deformation and a significant role of diffusion-based processes. Correspondingly, the thermal conductivity increases (Figure 8), reflecting reduced lattice, grain boundary, and pore scattering.

The contrast in densification behavior between the two powder sizes (Figure 7a) reflects likely differences in their packing behavior arising from particle morphology and surface characteristics. The 0.4 µm powder, having a higher surface area and stronger tendency to agglomerate, is likely packed less uniformly in the die, resulting in higher initial porosity (evident at 1000 °C in Figure 7a). As the temperature increases, pore closure proceeds first through enhanced plastic deformation and later through diffusion-driven mechanisms. The remaining difference in final density between 1800 °C and 2000 °C may also be influenced by variations in powder purity; the finer powder, due to its larger specific surface, contained more oxygen (see Section 2.1). While the total oxide content is below XRD detection limits, oxide particles were identified in the SEM images (Figure 3g,h). If 1% of the skeleton volume was WO_3_, this would result in a 0.6% difference in apparent porosity.

## 5. Conclusions

Tungsten compacts were prepared by hot pressing at an ultra-high pressure of 6 GPa, and the influence of starting powder size and sintering temperature was studied.

From the density/porosity results, one can infer that the finer powder was sintered to a lesser degree than the coarser one; this could be attributed to its tendency toward agglomeration and inhomogeneous settling in the die. The same trend was confirmed by the thermal conductivity measurements. While for the finer powder, density gradually increased with sintering temperature from ~90% to 96%, for the coarser powder, it remained practically independent, at around 98% of the theoretical value. Concerning the evolution of sintering degree with temperature, observations of the grain sizes (both by SEM and XRD) indicate a significant change only near the highest sintering temperature of 2000 °C. This is also corroborated by the hardness measurements. The hardness values, however, seem to be more correlated with the grain size than the degree of sintering or density. Tensile testing showed rather brittle behavior (indicated already by the cracking of some as-sintered samples) with strain to fracture in units of %. For the 2 µm powder, significant densification was observed already from the lowest temperatures, which is attributed to plastic deformation due to the high pressure.

In summary, the investigation showed the influence of both powder size and sintering temperature on the properties of the tungsten compacts. The positive influence of high pressure on densification was observed already at relatively low temperatures. However, the sintered materials exhibited rather brittle behavior. Therefore, under these conditions, the ultra-high pressure did not manifest a notable improvement in mechanical properties, compared to other sintering routes presented in the literature. For the potential usability of these materials in the harsh environment of fusion devices, further optimization of the process would be needed. Possible routes may include moderately increased pressure and variations in the powder sizes.

## Figures and Tables

**Table 1:** Characteristics of high-pressure sintering techniques and the resulting tungsten compacts. RSUHP = resistance sintering under ultra-high pressure, SPS = spark plasma sintering, PPC = plasma pressure compaction, HSC = hot-shock consolidation.

Method	Theoretical Density (%)	Sintering Temperature (°C)	Sintering Pressure (GPa)	Initial Powder Size (µm)	Grain Size (µm)	Citation
**RSUHP**	94.85–98.22	Not specified	9	0.2–7	Fine	[7]
**SPS**	90.8–94.3	1800–1300	0.09–0.266	~1	Multimodal	[8]
**PPC**	94–96	Not specified	0.8–1	0.1–0.275	Fine	[9]
**HSC**	92–96.7	1300–1054	3–4.3	2	~2	[10]

## Data Availability

Raw experimental data presented in this study are available on request from the corresponding author.

## Supplementary Materials

The following supporting information can be downloaded at: https://www.mdpi.com/article/10.3390/ma18235265/s1, Figure S1: XRD pattern of sample produced from 0.4 µm powder at 1000 °C; Figure S2: XRD pattern of sample produced from 0.4 µm powder at 1400 °C; Figure S3: XRD pattern of sample produced from 0.4 µm powder at 1800 °C; Figure S4: XRD pattern of sample produced from 0.4 µm powder at 2000 °C; Figure S5: XRD pattern of sample produced from 2 µm powder at 1000 °C; Figure S6: XRD pattern of sample produced from 2 µm powder at 1400 °C; Figure S7: XRD pattern of sample produced from 2 µm powder at 1800 °C; Figure S8: XRD pattern of sample produced from 2 µm powder at 2000 °C.
