# Supplementary material for: Characteristics of Tungsten Prepared by Hot Pressing at High Pressure
Source: Materials (Basel). 2025 Nov 21;18(23):5265. doi: 10.3390/ma18235265 (PMC12693080; doi:10.3390/ma18235265)
Supplement: Supplementary file 1 [file materials-18-05265-s001.zip › materials-3961245-supplementary.pdf]

materials

Article

Characteristics of Tungsten Prepared by Hot Pressing at High Pressure

Jiří Matějíček [1,*], Monika Vilémová [1], Andrii Rednyk [1], Hynek Hadraba [2], Zdeněk Chlup [2], František Lukáč [1], Romain Génois [1] and Jakub Klečka [1]

[1] Institute of Plasma Physics of the Czech Academy of Sciences, U Slovanky 1a, 18200 Praha, Czechia; vilemova@ipp.cas.cz (M.V.); rednyk@ipp.cas.cz (A.R.); lukac@ipp.cas.cz (F.L.); genois@ipp.cas.cz (G.R.); klecka@ipp.cas.cz (J.K.)
[2] Institute of Physics of Materials of the Czech Academy of Sciences, Žižkova 22, 61600 Brno, Czechia; hadraba@ipm.cz (H.H.); chlup@ipm.cz (Z.C.)
* Correspondence: matejicek@ipp.cas.cz

Academic Editor: Pan Gong

Received: 16 October 2025
Revised: 12 November 2025
Accepted: 19 November 2025
Published: date

Citation: Matějíček, J.; Vilémová, M.; Rednyk, A.; Hadraba, H.; Chlup, Z.; Lukáč, F.; Génois, R.; Klečka, J. Characteristics of Tungsten Prepared by Hot Pressing at High Pressure. *Materials* **2025**, 18, 5265. https://doi.org/10.3390/ma18235265.

Copyright: © 2025 by the authors. Submitted for possible open access publication under the terms and conditions of the Creative Commons Attribution (CC BY) license (https://creativecommons.org/licenses/by/4.0/).

1. Supplementary material – XRD patterns

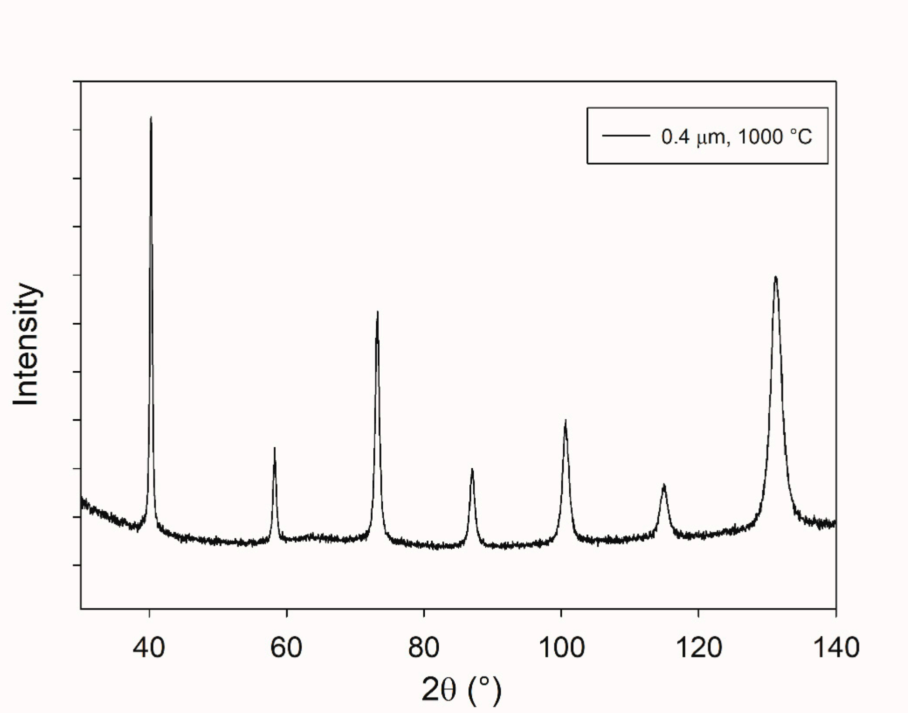

Figure S1. XRD pattern of sample produced from 0.4 µm powder at 1000 °C.

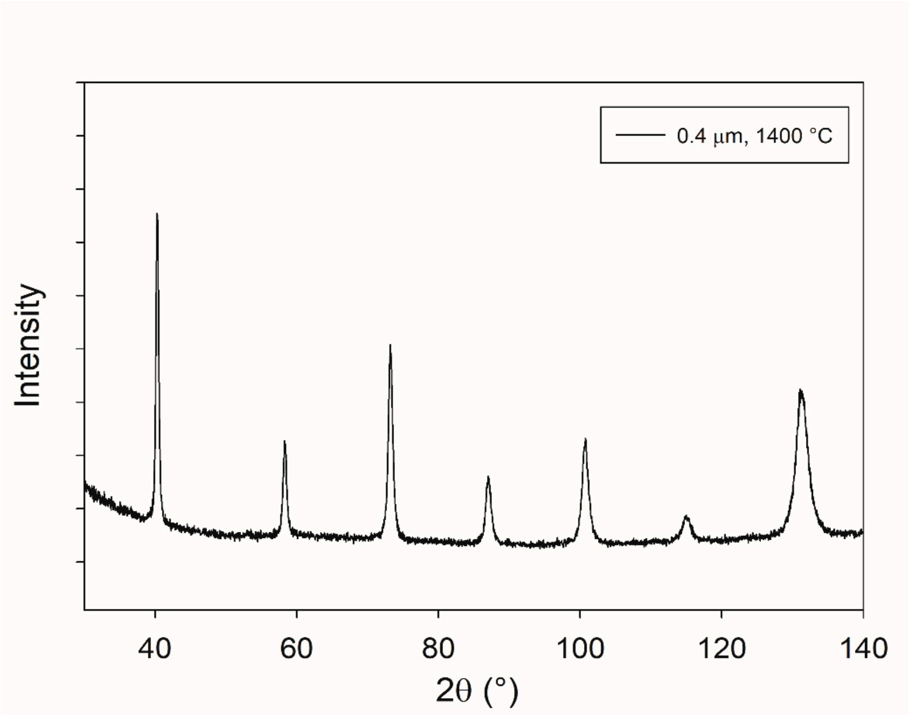

Figure S2. XRD pattern of sample produced from 0.4 μm powder at 1400 °C.

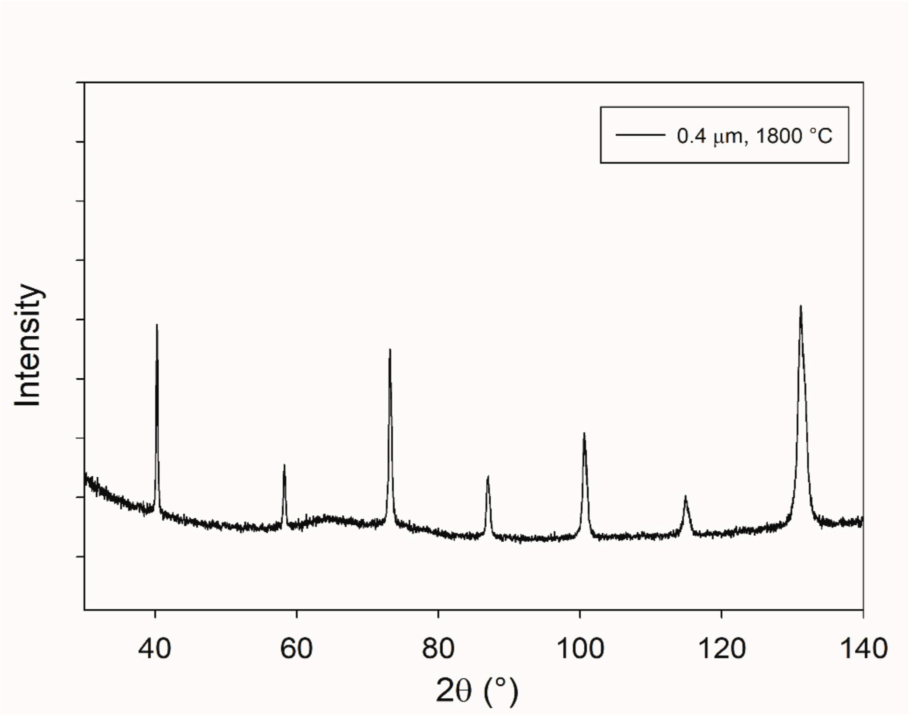

Figure S3. XRD pattern of sample produced from 0.4 μm powder at 1800 °C.

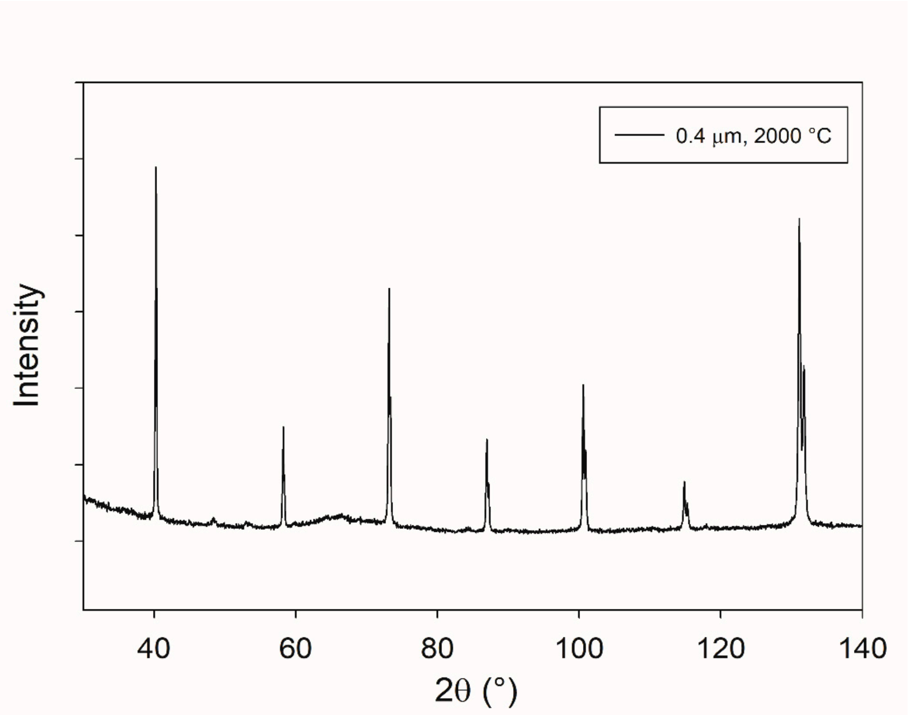

Figure S4. XRD pattern of sample produced from 0.4 µm powder at 2000 °C.

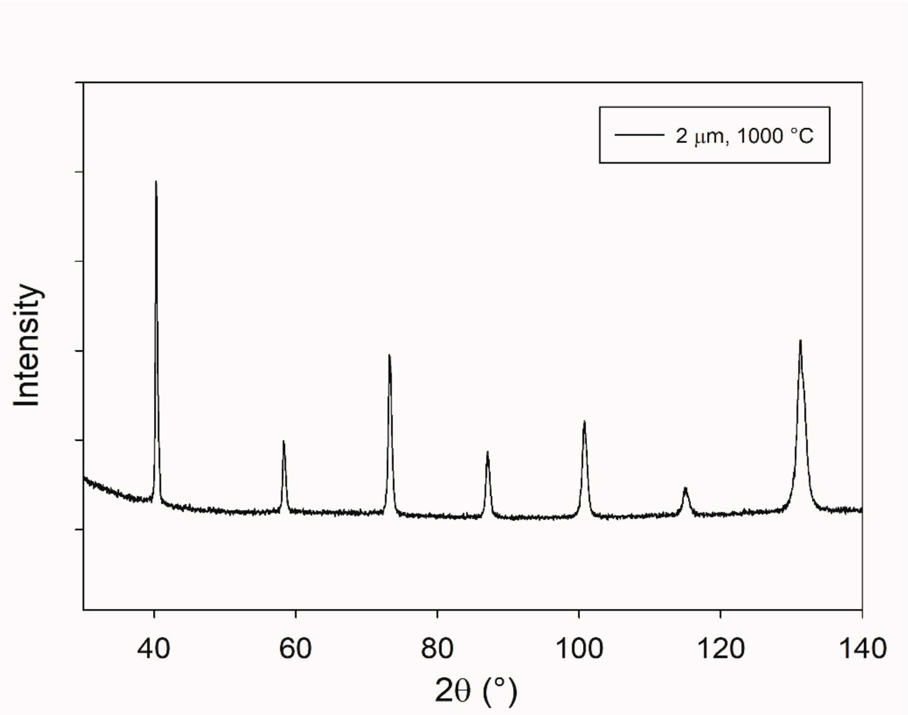

Figure S5. XRD pattern of sample produced from 2 µm powder at 1000 °C.

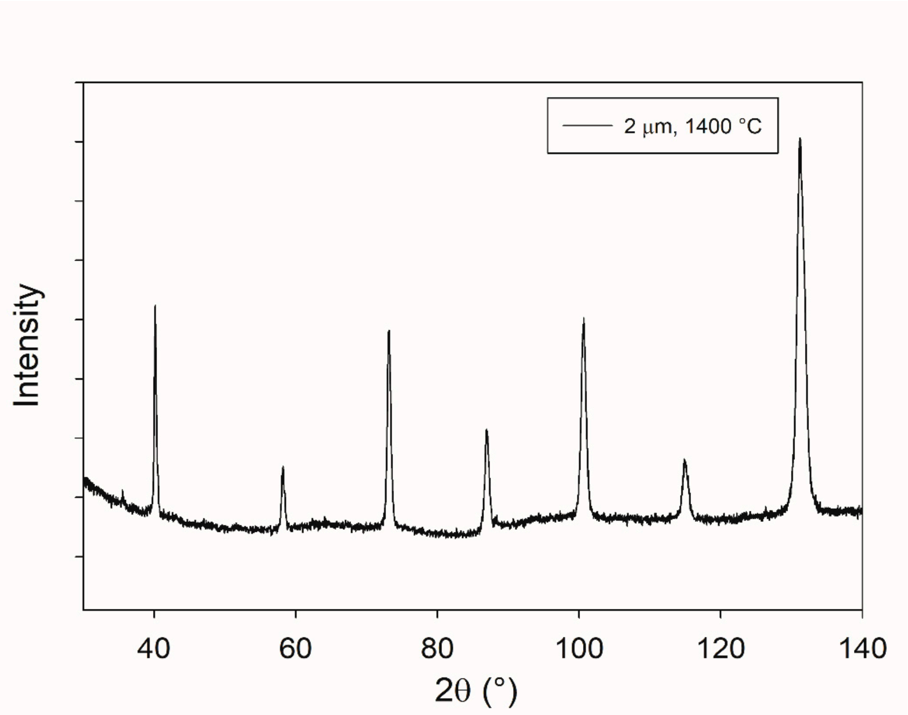

Figure S6. XRD pattern of sample produced from 2 µm powder at 1400 °C.

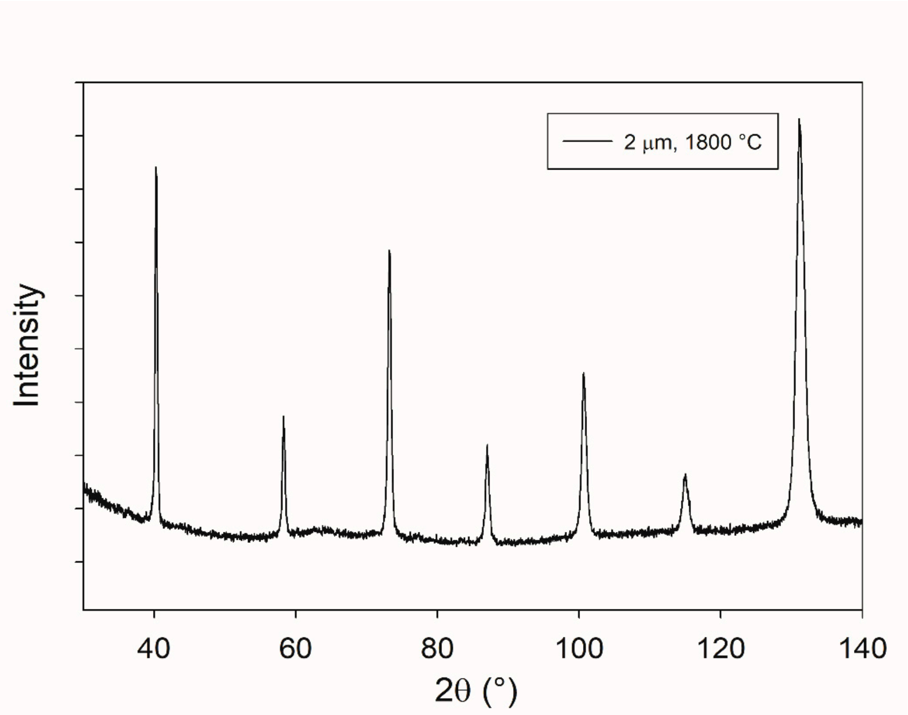

Figure S7. XRD pattern of sample produced from 2 µm powder at 1800 °C.

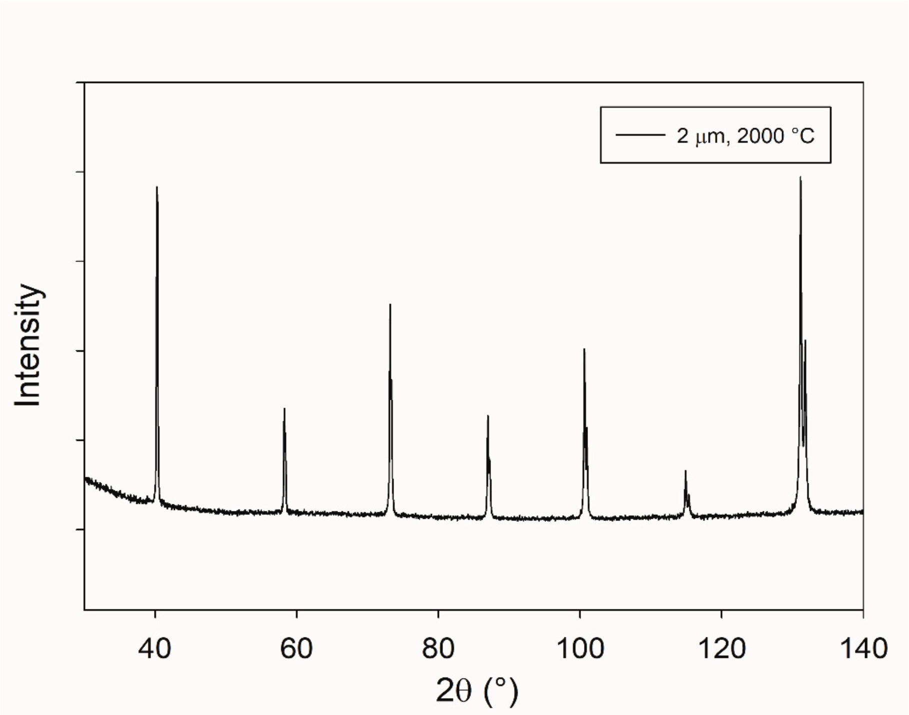

Figure S8. XRD pattern of sample produced from 2 µm powder at 2000 °C.
